# Aberrant Circulating Th17 Cells in Patients with B-Cell Non-Hodgkin’s Lymphoma

**DOI:** 10.1371/journal.pone.0148044

**Published:** 2016-01-26

**Authors:** Ting Lu, Shuang Yu, Yan Liu, Congcong Yin, Jingjing Ye, Zhi Liu, Daoxin Ma, Chunyan Ji

**Affiliations:** 1 Department of Hematology, Qilu Hospital, Shandong University, Jinan, PR China; 2 College of Information Science and Engineering, Shandong University, Jinan, PR China; European Institute of Oncology, ITALY

## Abstract

Non-Hodgkin’s lymphomas (NHLs) are a heterogeneous group of neoplasm in which 90% are B-cell lymphomas and 10% T-cell lymphomas. Although T-helper 17 (Th17) cells have been implicated to be essential in the pathogenesis of autoimmune and inflammatory diseases, its role in B-cell non-Hodgkin’s lymphoma (B-NHL) remains unknown. In this study, we observed a significantly decreased frequency of Th17 cells in peripheral blood from B-NHL patients compared with healthy individuals, accompanied with increased Th1 cells. IL-17AF plasma levels were remarkably decreased in B-NHL patients, accompanied with undetectable IL-17FF and unchangeable IL-17AA. Moreover, Th17 and Th1 cells became normalized after one or two cycles of chemotherapy. Interestingly, in B-NHL, circulating Th17 cells frequencies were significantly higher in relapsed patients than those in untreated patients or normal individuals. Meanwhile, there was no statistical difference regarding the frequencies of Th1 cells between relapsed and untreated patients. Taken these data together, circulating Th17 subset immune response may be associated with the response of patients to treatment and with different stages of disease.

## Introduction

T helper (Th) 17 cells are a subset of CD4+ effector T cells which uniformly express inflammatory chemokine receptor CCR6, and are characterized by expression of the interleukin (IL)-17 family cytokines including IL-17A and IL-17F [1–3]. Retinoic acid-related orphan receptor C (RORC) has been considered as the essential transcription factor for Th17 differentiation in human [4]. In recent decades, many studies have demonstrated that Th17 subset plays important roles in autoimmunity and inflammatory diseases such as experimental allergic encephalomyelitis, autoimmune arthritis, multiple sclerosis and psoriasis [5–8].

However, the specific role of Th17 cells in tumor is still uncertain and debatable. Results from two studies in advanced ovarian, pancreatic, renal cell carcinoma and uterine cervical cancer suggested that IL-17+ T cells would contribute to tumor pathogenesis [9, 10]. In contrast, another two researches showed that Th17 cells might play a beneficial role in ovarian cancer and prostate cancer [11, 12]. Moreover, the distribution of Th17 cells varied in different solid tumors or hematological diseases. Zou et.al showed that the highest levels of IL-17+ T cells were detected in tumor tissues in patients with advanced ovarian carcinoma and Cui et.al found a significantly increased circulating Th17 subset in uterine cervical cancer [9, 10]. Our previous studies have demonstrated that Th17 cells were significantly increased in peripheral blood from patients with acute myeloid leukemia (AML) and multiple myeloma (MM) while decreased in chronic myeloid leukemia (CML) [13–15]. However, little is known about circulating Th17 cells in lymphoma especially B-cell non-Hodgkin’s lymphoma (B-NHL).

IL-17A and IL-17F secreted by Th17 subset are 2 highly homologous pro-inflammatory cytokines and belong to IL-17 family which consists of six subtypes: IL-17A, IL-17B, IL-17C, IL-17D, IL17E and IL-17F [16]. Since their high degree of homology, IL-17A and IL-17F bind the same receptor complex which is comprised of IL-17RA and IL-17RC and consequently exhibits similar biological activities in many aspects [17, 18]. Both IL-17A and IL-17F can form disulfide-bonded IL-17AA, IL-17FF homodimers and IL-17AF heterodimer. Many studies have investigated the role of IL-17A, often referred to as IL-17, in inflammation, autoimmune disorders and tumors. Several studies have found higher expression of IL-17A in tumor tissues, such as multiple myeloma, ovarian cancers, gastric cancer and breast cancer [19–22]. Yet only several studies focused on IL-17F and IL-17AF. The mRNA expression level of IL-17F has been demonstrated increased in cutaneous T-cell lymphoma (CTCL) skin lesions and was also associated with progression of CTCL [23]. However, the expression of IL-17FF and IL-17AF in B-cell non-Hodgkin’s lymphoma remains undefined.

Non-Hodgkin’s lymphoma (NHL), a heterogeneous group of malignancies originating in lymphatic hematopoietic tissue, can be classified into B-cell lymphomas and T-cell lymphomas according to different types of lymphoid cells. B-cell non-Hodgkin’s lymphoma (B-NHL) is further classified into several subtypes. Among them, diffuse large B-cell lymphoma (DLBCL), follicular lymphoma (FL) and mantle cell lymphoma (MCL) are the most common subtypes of B-NHL [24, 25]. In this study, we examined the proportions of Th17 and Th1 cells and the concentrations of related cytokines (IL-17AA, IL-17AF, IL-17FF) in peripheral blood from patients with lymphoma especially B-cell non-Hodgkin’s lymphoma. In order to evaluate their involvement in pathogenesis and progression of patients with B-cell non-Hodgkin’s lymphoma, we also observed the frequencies of Th17 and Th1 cells in patients after treatment with chemotherapy or relapsed patients.

## Materials and Methods

### Patients and Controls

A total of 57 patients with lymphoma (21 females and 36 males, age range 18–79 years old, median 59 years old) were collected in this study. Collection took place from January 2013 to December 2013 at the Department of Hematology, Qilu Hospital, Jinan, China. All cases were consistent with lymphoma diagnostic criteria. The sources of patient-derived material and data are summarized in Table 1. Thirty-nine healthy controls (21 females and 18 males, age range 20–50 years old, median 32 years old) were included. Ethical approval for the study was obtained from the Medical Ethical Committee of Qilu Hospital, Shandong University. Written informed consent was obtained from all participants

**Table 1:** The detail information of patients in the current study.

Patients	Types	Age (year)	Gender		Subtypes	Numbers
			Male	Female		
Newly Diagnosed	B-NHL	52 (12.0)	16 (61.5)	10 (38.5)	DLBCL	17
					FL	4
					MCL	1
					CLL/SLL	2
					MZL	2
	T-NHL	55 (17.1)	8 (66.7)	4 (33.3)	ALCL	4
					AITL	8
	HL	27.5 (18.2)	7 (70)	3 (30)		10
Relapsed	B-NHL	49 (13.1)	5 (55.6)	4 (44.4)	DLBCL	6
					FL	1
					MCL	2
Totally						57

Ages are presented as median (SD) and genders are presented as n (%). B-NHL, B-cell non-Hodgkin’s lymphoma; T-NHL, T-cell non-Hodgkin’s lymphoma; HL, Hodgkin’s lymphoma; DLBCL, diffuse large B-cell lymphoma; FL, follicular cell lymphoma; MCL, mantle cell lymphoma; CLL/SLL, chronic lymphocytic leukemia/small lymphocytic lymphoma; MZL, marginal zone B-cell lymphomas; ALCL, anaplastic large cell lymphoma; AITL, Angioimmunoblastic T-cell lymphoma

### Treatment Regimens and Samples Collection

All lymphoma patients were treated with a standard induction chemotherapy based on newly NCCN guideline. All the 10 treated patients and 9 relapsed patients that were mentioned in our paper were treated with CHOP (cyclophosphamide, doxorubicin, vincristine, prednisone) regimen with or without rituximab. In order to avoid the drugs influence on the distribution of Th subsets, we collected blood samples from newly diagnosed patients before chemotherapeutic treatment. And the collection of blood samples from treated and relapsed patients took place after at least two weeks when they received chemotherapy.

### Flow Cytometric Analysis

Flow cytometry was used to study membrane makers and intracellular cytokines to identify the cytokine-producing cells. Briefly, heparinized peripheral blood (400 μl) with an equal volume of RPMI 1640 medium (Hyclone, USA) was incubated for 4 h at 37°C, 5% CO2 in the presence of 25 ng/ml of phorbol myristate acetate (PMA), 1 μg/ml of ionomycin, and 1.7 μg/ml monensin (all from Alexis Biochemicals, USA). After incubation, the cells were stained with Alexa Fluor® 647 or PerCP/Cy 5.5 anti-CD4 (isotype mouse IgG1, κ; clone RPA-T4) monoclonal antibody at room temperature in the dark for 20 min. After surface staining, the cells were next stained with FITC anti-IFN-γ (isotype mouse IgG1, κ; clone 4S.B3), PerCP/Cy 5.5 or PE anti-IL-17 (isotype mouse IgG1, κ; clone BL168) monoclonal antibodies after fixation and permeabilization. All the antibodies were from Biolegend (California, USA). Fixation and permeabilizaton reagents were purchased from eBioscience (California, USA). Isotype controls were given to enable correct compensation and confirm antibody specificity. Stained cells were analyzed by flow cytometric analysis using a FACS Calibur cytometer equipped with CellQuest software (BD Bioscience PharMingen, USA). For analysis, we first gated lymphocytes, then gated CD4+ T cells in lymphocytes, and analyzed the percentages of CD4+IL-17-producing (Th17) cells and CD4+IFN-γ-producing (Th1) cells in CD4+ T cells.

### Enzyme-Linked Immunosorbent Assay (ELISA)

Peripheral blood was collected into heparin-anticoagulant vacutainer tubes. All plasma specimens were obtained from all subjects by centrifugation and stored at −80°C for determination of cytokines. The concentrations of IL-17AA, IL-17FF and IL-17AF in each group were determined with a quantitative sandwich enzyme immunoassay technique in accordance with the manufacturer's recommendations (eBioscience, USA). The concentrations were calculated from a standard curve according to the manufacturer's protocol. Sensitive concentration of IL-17AA, IL-17FF and IL-17AF ELISA kit is 0.5 pg/ml, 15.5 pg/ml and 8.8 pg/ml respectively.

### Statistical Analysis

The results were expressed as median range. The Fisher’s exact test, Student t-test, Wilcoxon signed rank test, Mann-Whitney U test or Kruskal-Wallis test was applied to determine significant differences between groups. All tests were performed by GraphPad Prism 5.0 system. In these analyses, two-sided P-values <0.05 were considered to be statistically significant.

## Results

### Decreased Th17 cells in the newly diagnosed B-cell non-Hodgkin’s lymphoma neither T-cell non-Hodgkin’s lymphoma nor Hodgkin’s lymphoma

**Fig 1:**
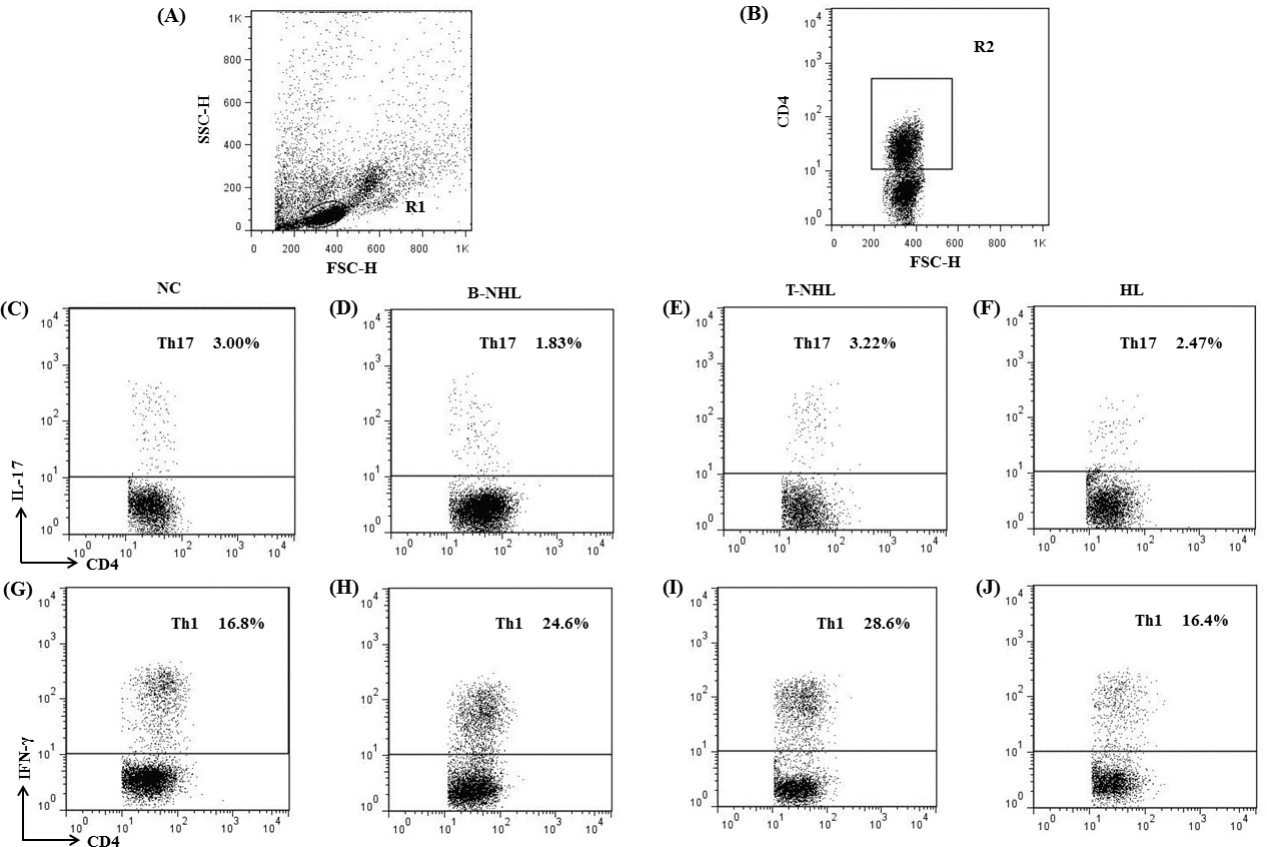
Dot plots of Th17 and Th1 cells. (A) Lymphocytes were gated in R1. (B) CD4+ T cells were gated in R2. (C-F) Representative FACS dot plots of Th17 (CD4+IL-17+) cells as a proportion of CD4+ T cells from normal controls (NC) and B-cell non-Hodgkin’s lymphoma (B-NHL), T-cell non-Hodgkin’s lymphoma (T-NHL), Hodgkin’s lymphoma (HL) patients. (G-J) Representative Th1 (CD4+IFN-γ+) cells as a proportion of CD4+ T cells from normal controls and B-NHL, T-NHL, HL patients.

**Fig 2:**
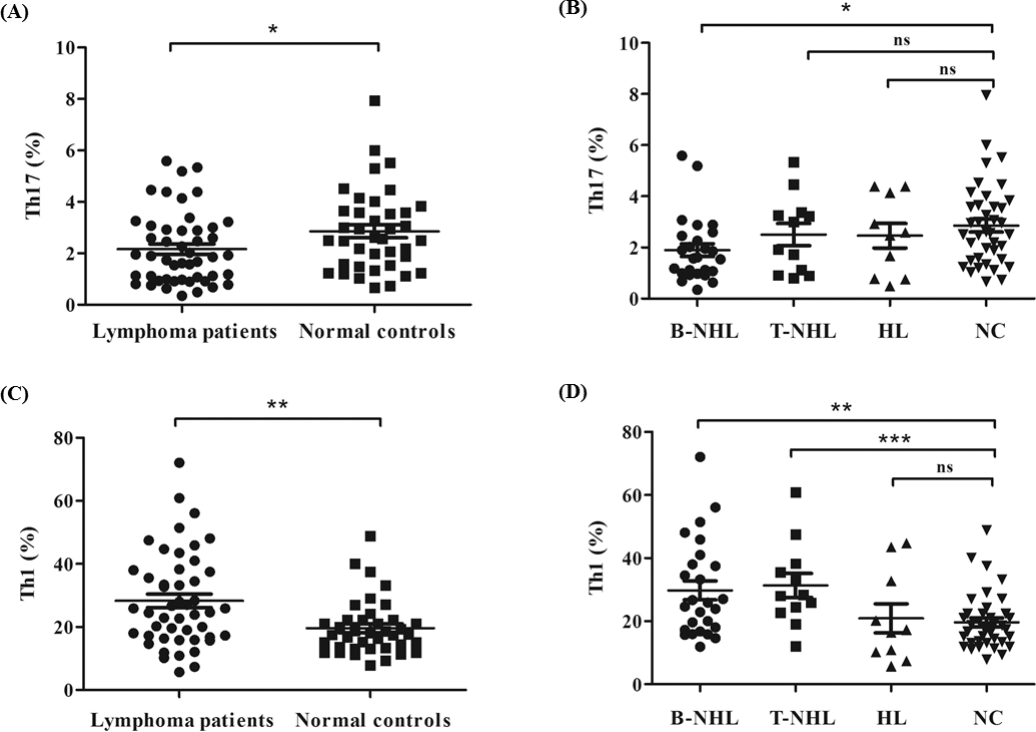
Results of Th17 and Th1 cells. (A) The percentage of circulating Th17 cells was significantly lower in lymphoma patients than normal controls (*P =* 0.0328). (B) Circulating Th17 cells were decreased significantly in patients with B-NHL (*P =* 0.0122) compared with NC while there was no significant difference in T-NHL (*P =* 0.4979) or HL (*P =* 0.4785). (C) The percentage of circulating Th1 cells was significantly higher in lymphoma patients than normal controls (*P* = 0.0030). (D) Circulating Th1 cells were statistically increased in patients with B-NHL (*P* = 0.0026) and T-NHL (*P* = 0.0008) rather than HL (*P* = 0.5852) compared to normal controls.* P<0.05; ** P<0.01; *** P<0.001; ns, non-significant

We analyzed the frequency of Th17 subset defined as CD4+IL-17-producing T cells after in vitro activation by PMA/ionomycin in short-term culture. The expression of a typical dot-plot of Th17 cells in representative patient and control was shown in Fig 1C–1F. Compared with healthy controls (median, 2.56% of CD4+ T cells; range, 0.66–7.95%; n = 39), the percentage of peripheral Th17 subset was significantly decreased in lymphoma patients (median, 1.88%; range, 0.35–5.59%; n = 48; *P* = 0.0328) (Fig 2A). Then we divided these lymphoma patients into three groups, B-cell non-Hodgkin’s lymphoma (B-NHL), T-cell non-Hodgkin’s lymphoma (T-NHL) and Hodgkin’s lymphoma (HL). The percentage of Th17 cells was statistically lower only in B-NHL (median, 1.59%; range, 0.35–5.59%; n = 26; *P* = 0.0122) neither T-NHL (median, 2.46%; range, 0.80–4.46%; n = 12; *P* = 0.4979) nor HL (median, 2.53%; range, 0.49–4.39%; n = 10; *P* = 0.4785) (Fig 2B). Meanwhile, there is no significant difference among these three different patient groups.

### Increased Th1 cells in the newly diagnosed non-Hodgkin’s lymphoma rather than in Hodgkin’s lymphoma

Since the developmental and functional features of Th17 cells were related to Th1 cells, we also analyzed Th1 frequencies in different groups of patients. The expression of a typical dot-plot of Th1 cells was shown in Fig 1G–1J. The proportion of CD4+IFN-γ-producing T cells (Th1 subset) was significantly higher in patients with lymphomas (median, 25.3% of CD4+ T cells; range, 5.7–72.1%; n = 48) than that in normal individuals (median, 17.6%; range, 7.9–48.9%; n = 39; *P* = 0.0030) (Fig 2C). Meanwhile, we further analyzed the percentages of Th1 cells in different patients groups and found that there was a remarkable increase in B-NHL (median, 25.3% of CD4+ T cells; range, 11.9–72.1%; n = 26; *P* = 0.0026) and T-NHL (median, 28.2%; range, 12.1–60.9%; n = 12; *P* = 0.0008) patients compared with those in normal controls (Fig 2D). However, no significant difference of Th1 was found between HL patients (median, 16.9%; range, 5.7–44.7%; n = 10; *P* = 0.5852) and healthy individuals (Fig 2D). And there was no statistical difference among these three different patient groups.

### Decreased IL-17AF with undetectable IL-17FF and non-changed IL-17AA in B-cell non-Hodgkin’s lymphoma

**Fig 3:**
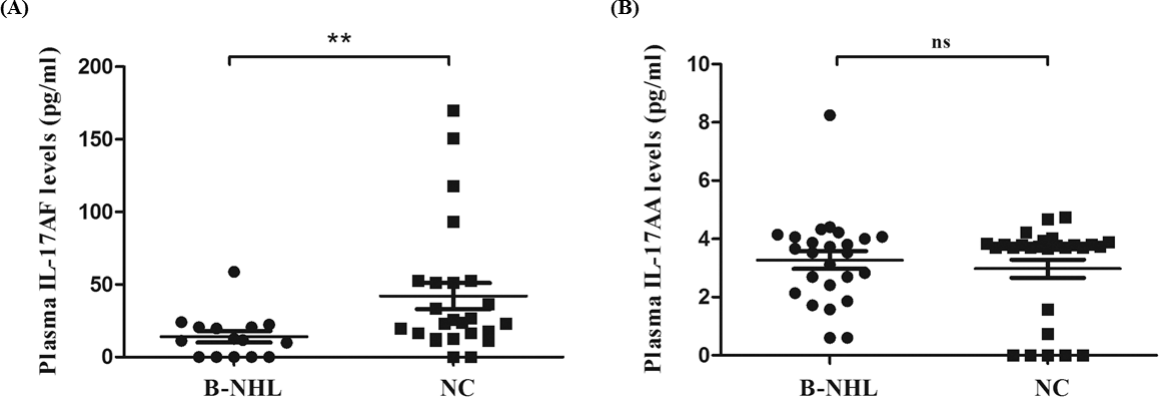
IL-17AF and IL-17AA concentrations in plasma from B-NHL patients and normal individuals. (A) IL-17AF levels in plasma from patients with B-cell non-Hodgkin’s lymphoma (B-NHL) were statistically decreased compared with normal controls (NC) (*P* = 0.0083). (B) IL-17AA concentrations in plasma of B-NHL patients were similar to normal individuals (*P* = 0.5010). ** P<0.01; ns, non-significant

Concentrations of IL-17AA, IL-17FF and IL-17AF in plasma from patients with B-cell non-Hodgkin’s lymphoma were measured by ELISA. We observed that IL-17FF could not be detected in plasma from neither patients with B-cell non-Hodgkin’s lymphoma or normal controls. And IL-17AF concentrations were significantly lower in patients (5 samples undetectable, median, 11.69 pg/ml; range, undetectable to 58.8 pg/ml; n = 15) than normal controls (2 samples undetectable; median, 23.69 pg/ml; range, undetectable to 169.92 pg/ml; n = 25; *P* = 0.0083) (Fig 3A). Meanwhile, there was no statistical difference regarding IL-17AA between patients (median, 3.52 pg/ml; range, 0.61 to 8.25 pg/ml; n = 25) and controls (5 samples undetectable; median, 3.74 pg/ml; range, undetectable to 4.74 pg/ml; n = 27; *P* = 0.5010) (Fig 3B).

### The relationship between Th17 or Th1 cells and the development of B-cell non-Hodgkin’s lymphoma patients

**Fig 4:**
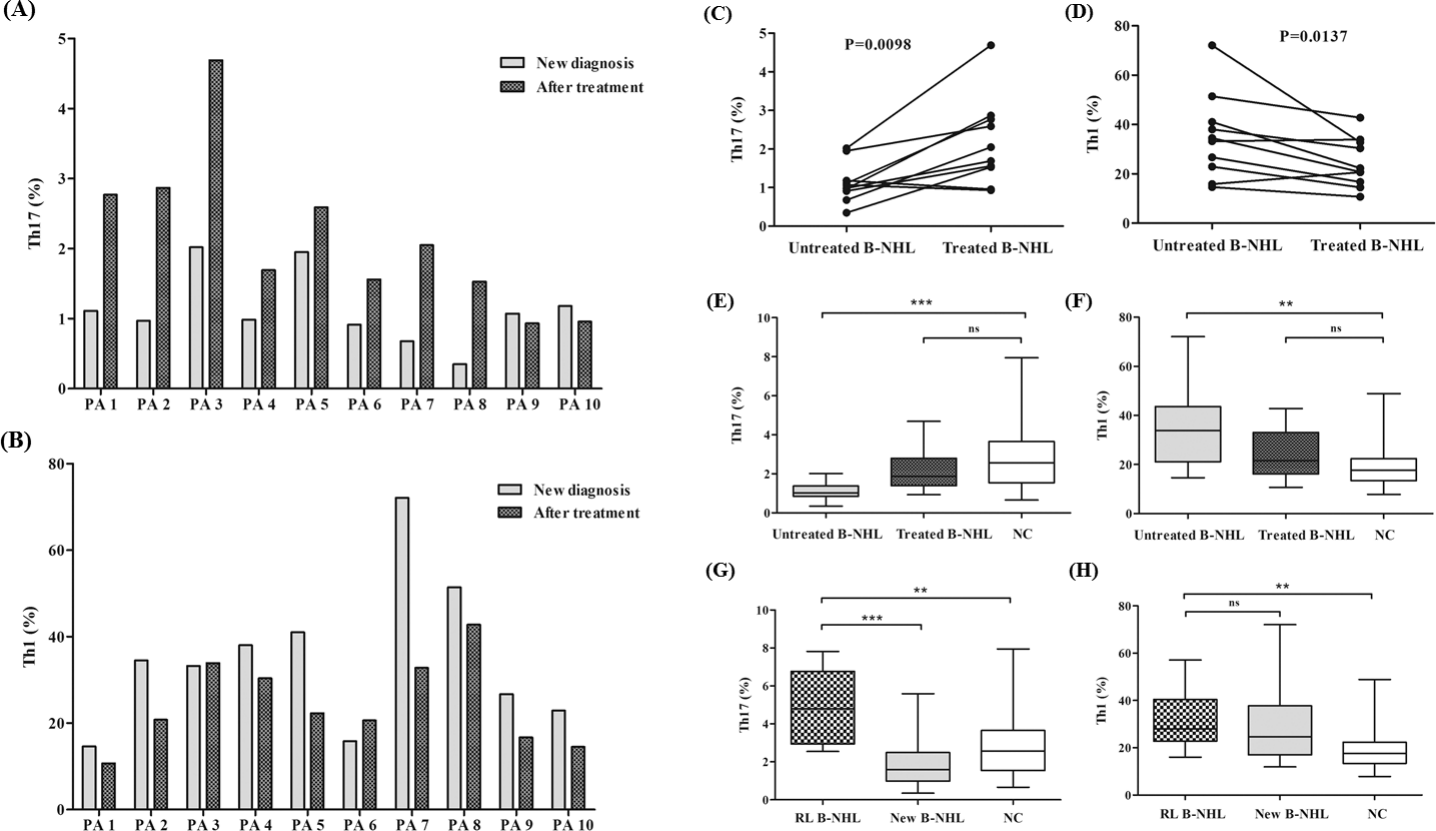
The percentages of circulating Th17 and Th1 cells in patients after treatment. PA means patient. (A and B) The percentages of circulating Th17 and Th1 cells before and after chemotherapy in B-NHL. (C and D) Circulating Th17 cells were significantly increased in treated B-NHL patients compared to those before treatment (*P* = 0.0098) while Th1 cells dramatically reduced (*P* = 0.0137). (E and F) Both Th17 and Th1 cells returned to normal values after treatment in B-NHL. (G) Circulating Th17 cells were dramatically elevated in relapsed patients (RL B-NHL) compared with new diagnosed patients (New B-NHL) (*P*<0.0001) and even normal individuals (NC) (*P* = 0.0015). (H) Circulating Th1 cells in relapsed patients were similar to new diagnosed patients (*P* = 0.7444) but still higher than normal individuals (*P* = 0.0014).). * P<0.05; ** P<0.01; *** P<0.001; ns, non-significant

Further to understand the influence of treatment on B-cell non-Hodgkin’s lymphoma microenvironment, we have detected Th17 and Th1 cells in 10 patients after treatment which was shown in Fig 4A and 4B. We found that the percentages of circulating Th17 cells were statistically increased in treated patients after one or two cycles of chemotherapy than those before treatment (*P* = 0.0098) while the percentages of Th1 cells decreased significantly (*P* = 0.0137) (Fig 4C and 4D). Both Th17 and Th1 cells were normalized after treatment (*P* = 0.2014, *P* = 0.1306) (Fig 4E and 4F).

We also collected other 9 relapsed B-cell non-Hodgkin’s lymphoma patients. Interestingly, compared with untreated patients and even healthy individuals respectively, a significant increase of peripheral Th17 was seen in relapsed patients (median, 4.79% of CD4+ T cells; range, 2.55–7.82%; n = 9; *P*<0.0001, *P* = 0.0015) (Fig 4G). Additionally, the percentages of Th1 cells in relapsed patients (median, 28.0% of CD4+ T cells; range, 16.0–57.1%; n = 9) were similar to those in newly diagnosed patients (*P* = 0.7444) but still significantly higher than normal controls (*P* = 0.0014) (Fig 4H).

## Discussion

Although Th17 cells have been intensively studied in autoimmune and infectious diseases, the specific role of Th17 cells in tumor is poorly understood. Recently, a few reports showed that the numbers of Th17 cells were increased in patients with solid tumors such as advanced ovarian carcinoma and gastric cancer [9, 20]. In hematological diseases, Wu et al. have described increased circulating Th17 cells in patients with AML [26] while Elisabeth et al. found normal Th17 cells in AML [27]. Besides, our previous studies observed increased circulating Th17 cells in MM but decreased in CML. Ansell et al. demonstrated that the frequencies of CD4+IL-17-producing T cells were significantly lower in biopsy specimens of B-NHL [28]. In the current study, we found that the percentage of Th17 cells was remarkably lower in circulating environment of patients with B-NHL neither T-NHL nor HL compared with that in normal individuals, which was consistent with Ansell et al.’ result.

Since the proximal cytokines governing Th1 cell development also potently suppressed Th17 development [29], we also detected the circulating Th1 frequency and found Th1 cells increased in B-NHL. Thus, Th1-promoting cytokine IFN-γ could inhibit the development of Th17 cells from naive precursors and neutralization of IFN-γ was necessary to enable development of IL-17 producing cells [30]. In a murine model study, reducing IFN-γ expression by anti-IFN-γ antibody resulted in the disappearance of the IL-17+IFN-γ+ T cells and an enhancement of IL-17+IFN-γ- T cells, suggesting a reciprocal regulation between development of Th1 and Th17 subsets [28]. However, several other studies have validated the Th17-Th1 transitions in models of chronic immune-mediated disease [31]. Taken these findings together, there may be also an overlap between the development of Th17 and Th1 in B-NHL as well that further investigation is needed.

By far, the impact of Th17 subset and associated cytokines on tumor development is still under debate. Tian et al. reported that abnormal Th17 frequencies were partially corrected after standard chemotherapy in AML [32]. Yin et al. found that imbalance of Th subsets occurred in peripheral blood of DLBCL patients during different chemotherapy stages and these Th subsets gradually return to normal values by 3 months after chemotherapy [33], suggesting that regulation of T lymphocyte subsets occurred in peripheral blood of DLBCL patients during different chemotherapy process. Similarly, after one or two cycles of standard chemotherapy, we found that both Th17 and Th1 cells were normalized in B-NHL. Furthermore, we detected the percentage of Th17 cells was dramatically increased in relapsed patients compared with untreated patients and healthy individuals while the percentage of Th1 cells in relapsed patients was similar to newly diagnosed patients. In colorectal cancer, Tosolini et al. demonstrated that patients with a low tumor-infiltrating Th17 response had a better disease-free survival [34]. On the contrary, Kryczek et al. observed decreased tumor-infiltrating Th17 response in advanced ovarian cancer, which was associated with a bad outcome [11]. Although multiple reports indicated that Th17 cells could promote anticancer immunity while others argued that these cells might exhibit tumor-promoting function, tumor progression is a complex process involving host-tumor interactions through multiple molecules of tumor microenvironment [34]. Several studies suggested that tumor microenvironments might selectively recruit Th17 cells and promote them trafficking from the periphery into tumor sites [35]. Our data showed that the changes of distribution of circulating Th17 cells were associated with the response of patients to treatment and with different stages of disease. This suggested that measurement of circulating Th17 cells might be related to tumor burden and be useful in evaluating therapeutic effect. Since the uncertain role of Th17 in tumors, we hypothesize that Th17 cells may play different roles in different stages of disease in B-cell non-Hodgkin’s lymphoma according to our data. However, further study focused on tumor-infiltrating Th17 in B-NHL should be performed to determine this hypothesis.

Th17 subset mainly produces IL-17A and IL-17F which can form homodimers or a heterodimer IL-17AF. A study documented that a number of malignant CTCL cell lines secreted IL-17A and IL-17F spontaneously. Given the right stimulus, these cells could also secrete IL-17AF. Their finding suggested that cytokines, antigens and inflammatory factors in local environment may regulate the expression of IL-17 cytokines in CTCL [23]. However, another two studies found that the presence of IL-17 showed opposite roles in proliferation and apoptosis of HL and cutaneous T-cell lymphoma cell lines [36, 37]. Here, our study observed normal IL-17AA in plasma of patients with B-NHL while the plasma levels of IL-17AF were dramatically decreased in patients compared with normal controls. And in neither patients nor normal individuals, the plasma IL-17FF was undetectable. However, IL-17AF has been little known in tumors, future studies should be considered.

In conclusion, we observed significantly decreased percentage of circulating Th17 cells in newly diagnosed B-NHL patients, along with decreased levels of plasma IL-17AF and increased circulating Th1 cells. Th17 and Th1 cells became normalized after chemotherapy while Th17 frequencies were dramatically increased in relapsed patients. These results indicate that the changes of distribution of circulating Th17 cells may be associated with the response of patients to treatment and with different stages of disease.
